# Gp78 E3 Ubiquitin Ligase: Essential Functions and Contributions in Proteostasis

**DOI:** 10.3389/fncel.2017.00259

**Published:** 2017-08-25

**Authors:** Vibhuti Joshi, Arun Upadhyay, Amit Kumar, Amit Mishra

**Affiliations:** ^1^Cellular and Molecular Neurobiology Unit, Indian Institute of Technology Jodhpur Jodhpur, India; ^2^Centre for Biosciences and Biomedical Engineering, Indian Institute of Technology Indore Indore, India

**Keywords:** Gp78, E3 ubiquitin ligases, neurodegenerative diseases, neurons, cancer

## Abstract

As per the requirement of metabolism and fitness, normal cellular functions are controlled by several proteins, and their interactive molecular and signaling events at multiple levels. Protein quality control (PQC) mechanisms ensure the correct folding and proper utilization of these proteins to avoid their misfolding and aggregation. To maintain the optimum environment of complex proteome PQC system employs various E3 ubiquitin ligases for the selective degradation of aberrant proteins. Glycoprotein 78 (Gp78) is an E3 ubiquitin ligase that prevents multifactorial deleterious accumulation of different misfolded proteins via endoplasmic reticulum-associated degradation (ERAD). However, the precise role of Gp78 under stress conditions to avoid bulk misfolded aggregation is unclear, which can act as a crucial resource to establish the dynamic nature of the proteome. Present article systematically explains the detailed molecular characterization of Gp78 and also addresses its various cellular physiological functions, which could be crucial to achieving protein homeostasis. Here, we comprehensively represent the current findings of Gp78, which shows its PQC roles in different physiological functions and diseases; and thereby propose novel opportunities to better understand the unsolved questions for therapeutic interventions linked with different protein misfolding disorders.

## Introduction

Glycoproteins are a special class of macromolecules composed of carbohydrate moieties attached covalently to proteins, thus achieving specialized structures to perform a variety of functions (Spiro, 1970). The N-glycosyl linkage between carbohydrate units and an asparagine residue, or O-glycosyl bonding with one of the serine, threonine or hydroxylysine residues of any cellular protein enables these molecular structures to play essential roles e.g., formation of membrane receptors, hormones, enzymes, immunoglobulins and lectins, etc. (Sharon, 1984; Shylaja and Seshadri, 1989). Autocrine motility factor receptor (AMFR), when binds with its ligand autocrine motility factor (AMF), induces a signaling cascade to mediate cancer cell motility and metastasis. It was later found that AMFR is a membrane-bound glycoprotein with molecular weight of 78 kDa, and hence termed as Gp78 (Liotta et al., 1986; Nabi and Raz, 1987). For a long time, AMFR remained a gene of high interest for cancer biologists, however, in last two decades, with increased interests of scientists in the field of cellular protein quality control (PQC) system and proteolytic machinery of the cell; AMFR became an important molecule for its involvement in the maintenance of cellular homeostasis.

Cells harbor an overcrowded heterogeneous population of macromolecules (Minton, 2000; Ellis, 2001), which posits a great challenge for the cellular system to regulate most of the processes with uttermost accuracy (Miyoshi and Sugimoto, 2008; White et al., 2010). Proteins, which perform most of the cellular tasks, remain prone to misfold and aggregate inside the cytoplasm to form large inclusion bodies, as have been reported to cause a number of neurodegenerative diseases (Soto, 2003; Soto and Estrada, 2008; Chhangani and Mishra, 2013). Therefore, to restrict such unwanted misfolding events, cells invest a huge amount of energy to provide an intracellular system to monitor, identify, repair and degrade any such proteinaceous inclusion bodies, generated inside the cell (Sharma et al., 2010). Chaperones are a class of proteins, which takes care of cellular proteins from very earlier stages of their synthesis and surveil them up to their degradation (Saibil, 2013). Some of them help the nascent polypeptide chains to fold co-translationally and achieve their native conformations with the greatest accuracy (Hartl et al., 2011). But, under various kinds of intra- and extracellular stresses, the working efficiency of chaperones is compromised, which adds up to the risks of misfolding and aggregation of proteins, several folds (Hipp et al., 2014). Under such conditions, other PQC components, i.e., ubiquitin proteasome system (UPS) and autophagy that in unison, form cellular proteolytic machinery, take over the charge and degrade intracellular bulk of aggregates to clear the cellular *milieu* (Chhangani et al., 2014).

E3 ubiquitin ligases are the key controllers of these triages. They are the specialized class of approximately 1000 different proteins (Nakayama and Nakayama, 2006), which maintain the turnover of cellular proteins under the normal basal conditions by tagging them with a small protein, ubiquitin, and direct them towards the 26S proteasome, for their degradation (Chen et al., 2011). E1 ubiquitin activating and E2 ubiquitin conjugating enzymes assist them in ubiquitination mechanism (Hershko and Ciechanover, 1992). At certain instances, they along with molecular chaperones, utilize lysosomal degradation machinery of the cell, by orchestrating a process called autophagy, to remove the bulk of the cellular inclusion bodies (Kuang et al., 2013; Upadhyay et al., 2015). These E3 ubiquitin ligases have been classified in different ways depending upon their structures and functions. Based on structural similarity, i.e., the presence of specialized domains, these proteins can broadly be classified into really interesting new gene (RING), homologous to E6-AP carboxyl terminus (HECT), U-box and plant homeodomain (PHD) domain containing E3 ubiquitin ligases (Metzger et al., 2012). Apparently, they could also be separated by their functional similarities. Quality control (QC) E3 ubiquitin ligases keep on monitoring and identifying any unwanted intracellular modifications in three-dimensional structures of the proteins, under various kinds of biotic and abiotic stress conditions; and by delivering them to cellular proteolytic systems they facilitate the degradation of these toxic inclusions formed inside the cells (McClellan et al., 2005; Chhangani et al., 2012).

Over the past few years, AMFR, the RING domain-containing E3 ubiquitin ligase, has been investigated for its crucial association with QC pathways, especially endoplasmic reticulum associated degradation (ERAD; Fang et al., 2001; Ying et al., 2009, 2011; Chen et al., 2012; Hara et al., 2014). A concerted action of E3 ubiquitin ligases, like AMFR, HMG-CoA Reductase Degradation 1 Homolog (Hrd1), Doa10; and ER resident chaperones, like immunoglobulin heavy chain-binding protein (Bip) and calnexin, helps in the correct folding of nascent polypeptides and retro-translocation of misfolded proteins from the ER lumen to the cytoplasm for their degradation (Mehnert et al., 2010; Christianson and Ye, 2014). Functional association of AMFR in cell signaling (Luo et al., 2002), metabolism (Watanabe et al., 1996), cell motility (Liotta et al., 1986); and regulatory control over cancer cell metastasis (Nabi et al., 1992), mitophagy (Fu et al., 2013) and ERAD are some major tasks, which this glycoprotein has been attributed so far. A plethora of studies has also been reported the association of AMFR in the proliferation of cells, tumor formation and maintenance (Silletti et al., 1993; Chiu et al., 2008). In last decade, attributing to its E3 ubiquitin ligase activity, roles of AMFR has also been investigated in various neurodegenerative disorders (Ying et al., 2009, 2011). Our review elaborates various such protective functions of AMFR and discusses possible regulatory control of this protein over various disease-associated pathways. We also provide a brief overview of the possible therapeutic strategies based on the applications of this E3 ubiquitin ligase in the cure of various diseases like cancer and neurodegeneration.

## Preview of Gp78: What Is History and Structural Impression and How It Is Differentially Distributed within Various Subcellular Locations?

Gp78 was initially reported as an intracellular intermediate of synthesis of viral glycoprotein Gp80, in rabies virus infected baby hamster kidney (BHK-21) cells (Madore and England, 1977). Few, years later, a group of scientists reported a membrane-bound glycoprotein with molecular mass of 78 kDa. They found that alterations in shape of metastatic cells lead to increased O-glycosylation of this glycoprotein Gp78, which enables it to participate in establishing interaction between cell and its external environment (Nabi and Raz, 1987). Further studies by the same group found that structural and functional characteristics, like surface localization and involvement in mediating cellular motility of melanoma cells, of Gp78 are similar to AMFR (Nabi et al., 1990). Experimental studies confirmed that Gp78 is the same surface bound molecule, which binds AMF and functions as its receptor to further mediate cell motility and metastasis of cancer cells (Nabi et al., 1991). Later, protein-protein binding assay analysis of AMF and cell surface glycoprotein also established AMF as a natural ligand of AMFR or Gp78 in B16-F1 melanoma cells (Silletti et al., 1991; Watanabe et al., 1991a).

Internalization of Type 1 membrane receptor, Gp78 and its ligand during metastasis is found to be associated with regulatory functions over cell kinesis (Watanabe et al., 1991b). Another study also confirmed that AMF and its binding with its receptor cause signal transduction to stimulate cell motility, same as chemotactic stimulation do in neutrophil mobility (Nabi et al., 1992). After observing these significant roles of Gp78 in metastasis, a group of researchers tried to reduce expression of this receptor to control tumor cell mobility (Lotan et al., 1992). It was found that the surface of carcinoma cells shows an increase in the population of AMFR, as compared to normal cells; however, in both type of cells, structure and copy number of the gene remains same (Silletti et al., 1993). In later years, several studies elaborated active involvement of AMFR in the maintenance of metastasis in various types of cancer cells (Nakamori et al., 1994; Otto et al., 1994; Silletti and Raz, 1996).

Human AMFR gene is located on chromosome 16; whereas in the mouse it is present on chromosome 8, with both the mRNA transcripts encode a protein of 643 amino acids in length (Shimizu et al., 1999; Chen et al., 2006). The N-terminus of the protein forms five transmembrane domains (Ponting, 2000), while C-terminus cytoplasmic tail contains most of the functional domains of the protein, e.g., RING finger domain, which is essentially required for Gp78 E3 ubiquitin ligase activity, spans from 340 to 382 amino acids (Song et al., 2005). RING finger motif comprises two histidines at fourth and fifth positions of the motif, termed as RING-H2 finger domain (Fang et al., 2003). The presence of the C-terminal RING finger motif also gives another designation to this molecule as a RING finger protein 45 (RNF45; Fairbank et al., 2009; St-Pierre et al., 2012). Another important domain necessary for interaction with Ubc, E2 ubiquitin conjugating enzymes, is a coupling of ubiquitin conjugation to the ER degradation (CUE) domain, which is located towards C-terminus of the RING finger (Ponting, 2000; Song et al., 2005). The similarities, observed by analysis of domains and functions of Gp78 with yeast E3 ubiquitin ligase Hrd1p and its cofactor Cue1p, suggest an evolutionary relationship between these two proteins (Chen et al., 2012). The hydrophobic segment of the cytosolic domain of Gp78 is one among the two oligomerization sites (OS) and form hetero-oligomer with its E2 conjugating enzyme (Li et al., 2009).

**Figure 1:**
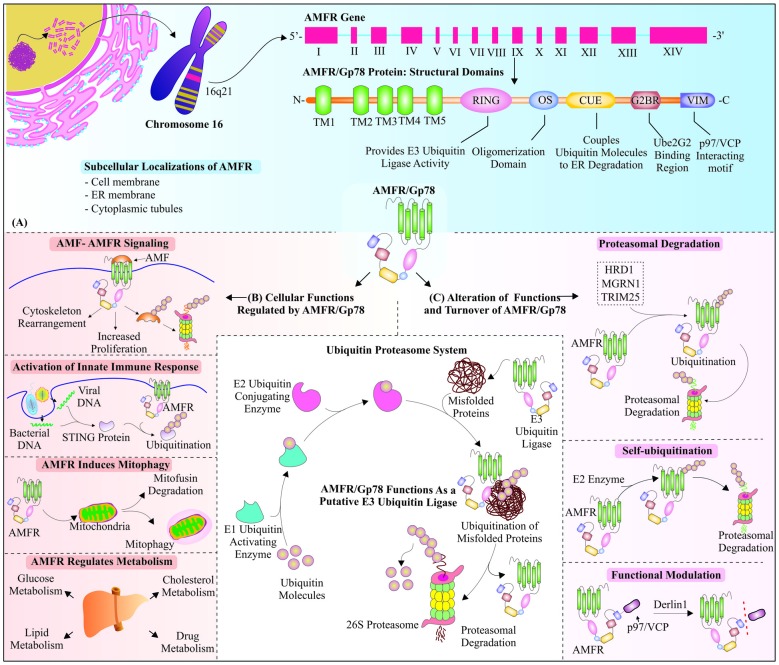
Overview of structure, functions and regulations of autocrine motility factor receptor (AMFR)/Glycoprotein 78 (Gp78): Membrane-bound receptor, as well as really interesting new gene (RING) E3 ubiquitin ligase AMFR/Gp78, is encoded by AMFR gene, which is located at chromosome number 16. AMFR/Gp78 is known to participate in various cellular pathways and is itself regulated in multiple ways. **(A)** The mRNA coding for AMFR/Gp78 consists of fourteen exons (Tsai et al., 2012), which form a mature protein with five transmembrane domains at N-terminus, followed by other functional domains, *viz*., RING, an oligomerization domain, Coupling of ubiquitin conjugation to the ER degradation (CUE), G2BR and Valosin-interacting motif (VIM). **(B)** Gp78 is involved in different cellular pathways, such as signaling, innate immune response, metabolism and induction of mitophagy. **(C)** The turnover of Gp78 is regulated either by E3 ubiquitin ligase activities of Hrd1, mahogunin ring finger 1 (MGRN1) and tripartite motif-containing protein 25 (TRIM25); or it could ubiquitinate itself in the presence of E2 conjugating enzymes. AMFR is functionally modulated by endoplasmic reticulum associated degradation (ERAD) E3 ubiquitin ligase Derlin 1. In the center, we have shown a general overview of ubiquitin proteasome system (UPS).

Further structural analysis of C-terminus of AMFR protein identified another region, called Ube2G2 binding region (G2BR), which comes into play for binding of AMFR with Ube2G2, an E2-conjugating enzyme (Chen et al., 2006). The interaction of Ube2G2: G2BR domain brings conformational alterations in E2 ubiquitin conjugating enzyme and increases the affinity of Ube2G2 for AMFR/Gp78 (Das et al., 2009). The study of Gp78 and its binding with E2 conjugating enzyme Ube2G2 confirms the two oligomerization sites, among which hydrophobic site is present towards the cytosolic domain of Gp78. The hetero-oligomer formed by Gp78 and Ube2G2 enables other Ube2G2 molecules to come closer, which provide easy transfer of ubiquitin molecules to nearby E2s that lead to active site-linked polyubiquitin chains (Li et al., 2009). Valosin-containing protein (VCP)-interacting motif (VIM) is the last domain, which is present on distant C-terminus of the protein and is crucially required for interaction of AMFR with AAA ATPase p97 enzyme, and its cofactor Ufd1-Np14, during ERAD (Ballar et al., 2006). Interestingly, a subsequent study demonstrated that Ufd1 (ubiquitin fusion degradation 1) might bind to AMFR without VCP (Cao et al., 2007). Figure 1A represents the structural overview of AMFR mRNA and protein with descriptive arrangements of its functional domains.

Fluorescence microscopic analysis revealed that apart from its presence over the cell surface, AMFR is also distributed throughout the cytoplasm in vesicular and tubular structures (Benlimame et al., 1998). Perinuclear and peripheral distribution of AMFR tubular structures was also observed through electron microscopy, which gets disrupted by the disruption of microtubular organization of the cells (Benlimame et al., 1995). Moloney sarcoma virus (mos)-transformed MDCK (MSV-MDCK) cells have shown concentrated AMFR tubules at the pericentriolar region of microtubules, further confirming AMFR with cell motility-related functions (Nabi et al., 1997). Ilimaquinone, a drug known for its Golgi-vesiculation abilities, may also disrupt AMFR tubules; and the morphological similarities of fragmented tubules with smooth ER suggests that these tubules could be the subdomains of smooth ER (Wang et al., 1997). Electron microscopic studies revealed that a small fraction of AMFR along with its ligand AMF could also be localized in cell-surface caveolae, which are recycled between surface and ER by internalization and trafficking to ER membranes via clathrin-independent endocytic pathways (Benlimame et al., 1998). However, in later years, it has been found that AMFR may also be endocytosed through MVBs and are recycled even after microtubule disruption and inhibition of endocytosis, showing a possible mechanism of AMFR internalization in a clathrin-dependent manner (Le et al., 2000).

## Is Gp78 A Promising E3 Ubiquitin Ligase? Optimum Regulation of Different Cellular Functions Under Crucial Conditions

The most studied and well-known function, for which AMFR is known, is its involvement in the motility and metastasis of different types of cancer cells (Liotta et al., 1986; Nabi et al., 1990; Watanabe et al., 1991b). The ligand-receptor binding of AMF-AMFR regulates multiple signaling processes and hence affects cell growth, motility and the programmed cell death apoptosis (Yanagawa et al., 2004). Overexpression of Gp78 is not only involved in progression or mobility of cancer cells, but it has also been found that in NIH3T3 cells, it induces transformation; whereas, in nude mice, enhanced expression of this molecule produces tumor (Onishi et al., 2003). Recent studies have also explored the involvement of AMFR E3 ubiquitin ligase activity in mounting innate immune responses inside the cells by polyubiquitinating and modifying functions of the stimulator of interferon genes (STING), which senses the foreign genetic materials and responds by triggering the production of interferon proteins (Wang Q. et al., 2014).

Selective mitochondrial degradation occurs inside the cells to remove old and defective mitochondria, and AMFR regulates this process by targeting mitochondrial proteins, mitofusins, for proteasomal degradation, inducing mitochondrial fragmentation (Fu et al., 2013). Purification and microsequencing of AMF demonstrated it as neuroleukin and enzyme phosphohexose isomerase, which catalyzes glucose 6-phosphate to fructose 6-phosphate isomerization, thus playing an indispensable role in glycolysis (Watanabe et al., 1996). Apart from glycolytic pathway, AMFR is also found to be implicated in the regulation of lipid and cholesterol metabolic pathways (Timar et al., 1993; Song et al., 2005; Liu et al., 2012). AMFR also facilitates the ubiquitination and degradation of cytochrome P450s of the 3A subfamily 4 (CYP3A4), a major enzyme involved in drug metabolism, taking place inside the liver, and also has a crucial regulatory control over metabolism of drugs (Wang et al., 2012). Gp78 as an E3 ubiquitin ligase, mainly participate in the degradation of ERAD substrates, and it is well explored, once CD3-δ was established as its putative ERAD substrate protein (Fang et al., 2001). Apart from playing these crucial roles inside the cells (as shown in Figure 1B), AMFR has also been reported for a plethora of roles and responsibilities in various pathways and diseases, which we will discuss in further sections of the review.

Cells regulate the level and functions of AMFR very precisely by multiple mechanisms so that a static level of AMFR could be maintained. The most important mechanism used for this is targeting of AMFR by other ER-resident E3 ubiquitin ligase Hrd1 or synoviolin, which ubiquitinates and degrades it in a proteasome-dependent manner (Shmueli et al., 2009). Similarly, investigation of other E3 ubiquitin ligases involved in ERAD, suggests that tripartite motif-containing protein 25 (TRIM25), which assists Gp78 in polyubiquitination of AMF, also participates in maintaining a steady-state level of Gp78 by its ubiquitination and degradation (Wang Y. et al., 2014). Recently, Gp78 was also identified as a substrate of mahogunin RING Finger 1 E3 ubiquitin ligase. Under normal cellular conditions MGRN1 ubiquitinates Gp78 at the K11 position and degrades it to regulate mitophagy; however, in mitochondrial stress condition, the cytosolic level of calcium increases, which interferes with the interaction of these two ligases (Mukherjee and Chakrabarti, 2016). Self-ubiquitination is another interesting way of regulating cellular levels of protein, which is an interesting feature found in many RING finger-containing E3 ubiquitin ligases (Metzger et al., 2014). Gp78 has also shown similar RING-dependent self-ubiquitination, through binding with E2 enzymes Ube2G2 and Ubc7, via its G2BR domain, and transfering ubiquitin molecules with the assistance of CUE domain (Fang et al., 2001; Chen et al., 2006). Another way to regulate the functionalities of this ER-resident E3 ubiquitin ligase is its Derlin1-mediated functional inhibition by uncoupling of p97/VCP and Gp78 (Ballar et al., 2007). A schematic overview of all these mechanisms, controlling the functions and turnover of AMFR has been represented in Figure 1C.

## Endoplasmic Reticulum Associated Degradation (ERAD) Linked E3 Ubiquitin Ligase Gp78: A Strong Early Defender Against Aberrant Proteins Accumulation

**Figure 2:**
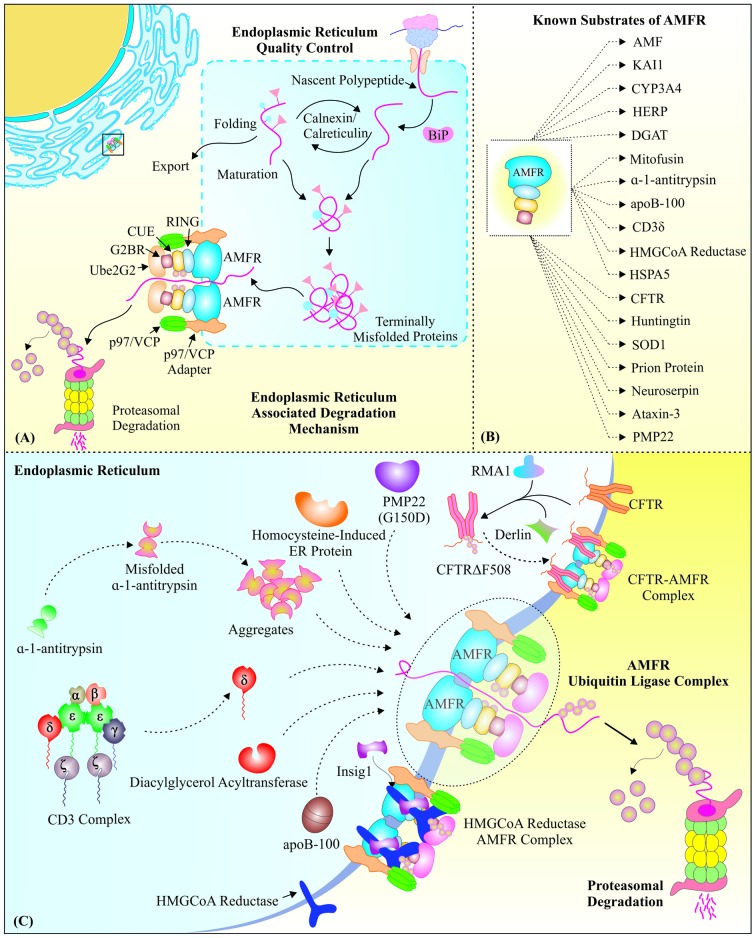
Gp78, as a vital E3 ubiquitin ligase of endoplasmic reticulum-associated degradation: AMFR/Gp78 is a crucial component of ERAD, the special Protein quality control (PQC) system of the endoplasmic reticulum. **(A)** The figure represents a basic mechanism of protein folding, misfolding and targeting of these misfolded proteins for proteasomal degradation from the lumen of endoplasmic reticulum (ER), with the aid of specific ER chaperones and E3 ubiquitin ligases like Gp78. **(B)** Summary of substrates regulated by Gp78 E3 ubiquitin ligase function. **(C)** A mechanistic overview of how Gp78 mediates retro-translocation of various ER proteins, like mutated CFTR, CD3-δ, etc., for their proteasomal degradation, to maintain the ER proteostasis and reduce the state of ER stress.

Cellular proteins reside in various compartments of the cells, at different stages of their lifespan. Nascent polypeptides are subjected to the endoplasmic reticulum (ER) for their maturation and post-translational modifications (Braakman and Bulleid, 2011). In doing so, they always remain prone to misfolding events (Hebert and Molinari, 2007). Therefore, to avoid accumulation of such obnoxious non-functional proteins inside ER; membrane of ER is equipped with a complex of proteins, which is capable of translocating these aberrant proteins from ER lumen to the cytoplasm (Tsai et al., 2002). These excluded toxic elements are later degraded by cytoplasmic QC components, like 26S proteasome (Kopito, 1997). A brief overview of ERAD components and the associated mechanism has been presented in Figure 2A, to provide a better understanding of this pathway.

Gp78, a RING finger domain containing protein, is also located on ER membranes and has shown E3 ubiquitin ligase-like activity. We have summarized various known substrates of Gp78 E3 ubiquitin ligase activity in Figure 2B. Gp78 along with MmUBC7, an E2 conjugating enzyme, degrades CD3-δ specifically from the CD3 complex, the T-cell antigen receptor (Fang et al., 2001). As stated earlier, the process of ERAD involves retro-translocation of misfolded proteins from ER to the cytosol. To carry out this process, AMFR needs to associate with AAA ATPase p97/VCP by a unique VIM; however, siRNA-based knockdown studies suggested that Gp78 may also degrade its ERAD substrates by an Ufd1-independent pathway (Ballar et al., 2006). The presence of Gp78 at ER membrane allows it to work in complex with other components of ERAD pathway to identify and exclude misfolded proteins inside the ER lumen (Zhang et al., 2015b). Transfection studies on HepG2 cell and cell-free system confirm that the components of low-density lipoproteins (LDL) and very low-density lipoproteins (VLDL), apolipoprotein B, is ubiquitinated in a VCP-dependent manner by the ER-resident E3 ubiquitin ligase Gp78 and is degraded through proteasome (Liang et al., 2003; Fisher et al., 2008).

Gp78, apart from being an E3 ubiquitin ligase, may also exhibit E4 like activity, as has been found in ERAD of a mutant form of cystic fibrosis transmembrane conductance regulator (CFTR∆F508), where it recognizes already conjugated ubiquitin molecules to substrate protein mediated by another upstream E3 ubiquitin ligase Ram 1 homolog (RMA1; Vij et al., 2006; Morito et al., 2008). Hrd1 mediated ubiquitination of Gp78 causes inhibition of CFTR∆F508 degradation; similarly, gene silencing of Gp78 by RNAi or its inhibition by small p97/VCP interacting protein (SVIP) also results in accumulation of this mutant protein (Ballar et al., 2010). α-1-antitrypsin, the serine proteinase inhibitor that protects tissues from an attack of neutrophil elastase, generally have a Z mutation (Glu 342 Lys); and this mutant form is ubiquitinated by Gp78 in conjugation with a mammalian Ubc7 E2 enzyme, and translocated to the cytoplasm and degraded by the proteasome (Shen et al., 2006).

Diacylglycerol acyltransferase isoform 2 (DGAT2), an enzyme involved in the synthesis of triacylglycerol, interacts directly with Gp78 for polyubiquitination and proteasomal degradation (Choi et al., 2014). The process of ERAD and stability of E3 ubiquitin ligases involved in this process is differentially regulated by the ER stress, as it increases the stability of Gp78, with no significant effect on the level or stability of Hrd1 (Shen et al., 2007). The interaction of AMFR and its ligand AMF also provides protection in ER stress condition, by regulating ER calcium release in the cytosol (Fu et al., 2011). ER stress-induced homocysteine-induced ER protein (HERP) is recently identified as proteasomal degradation substrate of Ube2g2–gp78-complex (Yan et al., 2014). The *in vivo* study, carried out in zebrafish, for Gp78 expression levels during ER stress, indicates its protective functions against ER stress in liver (Chen et al., 2014). Descriptive schematic of the roles of Gp78 in ERAD degradation pathways of various substrates is drawn in Figure 2C.

## Neurobiological Functions of Gp78: Impairment May Lead to Protein Aggregation and Neurodegeneration

E3 ubiquitin ligases play an important role in overall development and maintenance of a healthy set of neurons throughout life, since early neonatal periods and up to the late onset neurodegenerative changes taking place in our brain (Upadhyay et al., 2017). In recent years, few studies have been done to investigate the roles of Gp78 in the development of the brain. The study performed on rat cerebellum spotted higher expression level of AMFR at postnatal state in comparison with an adult, showing the probable role of AMFR in granule cells migration; however, localization study showed AMFR expression in neurites, cell body and growth cones of neurons to regulate neuroleukin activities (Leclerc et al., 2000). A novel role of AMFR has also been postulated in strengthening the learning and establishing memory, as hypothesized by increased expression of the AMFR in hippocampus region that is also affirmed by conducting several tests on rats and mice (Luo et al., 2002; Yang et al., 2012). There are very limited number of studies, which have been done to establish a direct link between Gp78 and neurodevelopment. Still, many groups have shown its involvement in neuroprotection against various stresses generated by inclusions formed of several disease-associated proteins.

The E3 ubiquitin ligase activity of AMFR has given it a considerable importance in recent past for its implication in a number of neurodegenerative diseases. The involvement of Gp78 in neuroprotection came into existence with studies based on disease-associated aggregatory proteins superoxide dismutase-1 (SOD1) and ataxin-3, which are targeted by Gp78 for ubiquitination and proteasomal degradation (Ying et al., 2009). Another study shows that expanded polyglutamine-containing huntingtin protein interacts with CUE domain of Gp78 and this interaction interferes with the interaction of Gp78 and ER chaperones, causing increased ER stress. However, Gp78 ameliorates such obnoxious condition by ubiquitinating and degrading the mutant huntingtin through ERAD (Yang et al., 2010). The neurodegenerative disorder familial encephalopathy occurs due to inclusion body formation by secretary glycoprotein neuroserpin, mainly in ER of neurons (Miranda et al., 2004). Hrd1 and Gp78 were identified as ERAD E3 ubiquitin ligases, which polyubiquitinate mutated forms of neuroserpin and target them for degradation in the association of VCP to abrogate toxicity, generated by their aggregates inside cortical and sub-cortical neuronal population (Ying et al., 2011).

Bovine spongiform encephalopathy and Creutzfeltd-Jacob diseases are other forms of neurodegenerative diseases, which are caused by a special class of proteins, called prions (PrP; Prusiner, 1991). Human prion protein could be present in multiple forms inside the cells, and unglycosylated forms of PrP has a critical association with PrP aggregates (Taraboulos et al., 1990). ER-resident Gp78 specifically interacts with C-terminal region of unglycosylated prion proteins, and ubiquitinate them for their degradation in proteasome-dependent manner (Shao et al., 2014). Mutation in the peripheral myelin protein 22 (PMP22) and its accumulation in endoplasmic reticulum gives rise to Charcot-Marie-Tooth (CMT) disease, a common peripheral nervous system disorder (Roa et al., 1993). Among different mutant forms of this protein, Gp78 degrades disease-causing mutated form PMP22 (G150D) via proteasomal pathway (Hara et al., 2014).

**Figure 3:**
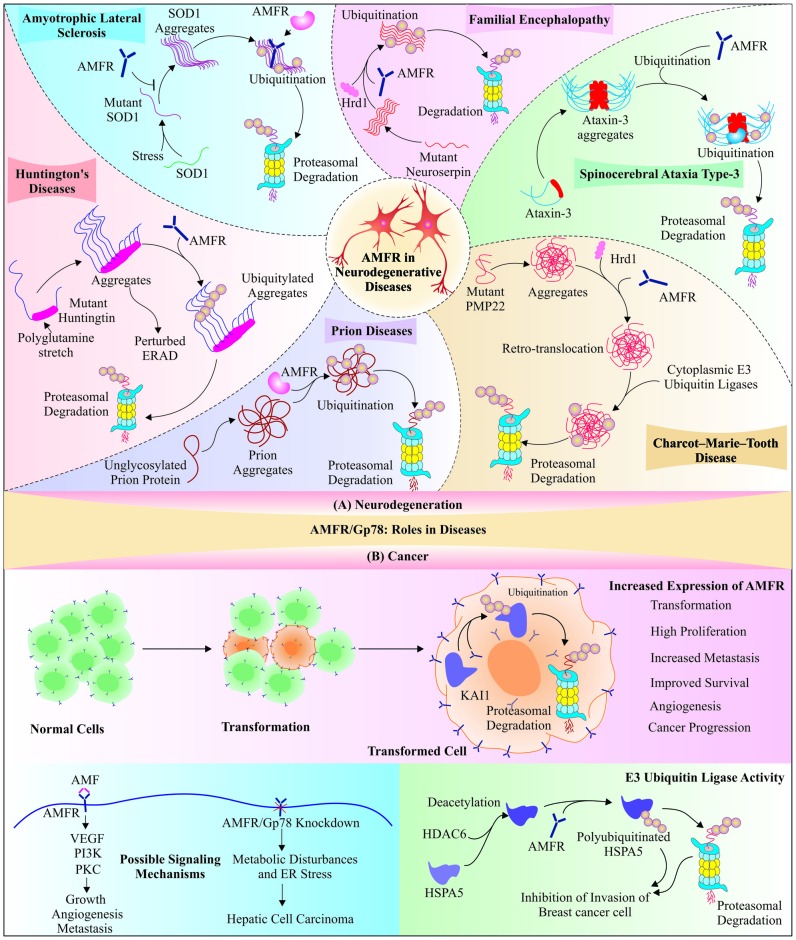
Functional implications of Gp78 in neurodegeneration and cancer: The partially explored roles of Gp78 in various diseases, like neurodegeneration and cancers, propose its therapeutic significance. **(A)** An E3 ubiquitin ligase function of Gp78 is well explored in neuroprotection as it facilitates the degradation of different kinds of aggregatory proteins, which are directly involved in neurodegenerative disorders; including amyotrophic lateral sclerosis (ALS), familial encephalopathy, cerebrospinal ataxia type-3, Charcot-Marie-Tooth (CMT) disease, prion diseases and Huntington’s disease. **(B)** Miscellaneous association of Gp78 was observed in cancer: Gp78 regulates transformation, invasion, and metastasis of tumor cells. Contrary to this, it suppresses invasion of breast cancer cells by degrading heat-shock protein 5 (HSPA5), which is deacetylated by histone deacetylase-6 (HDAC6). The Gp78 knockdown mice developed hepatic cell carcinoma because of disturbed metabolism, which also proposes tumor suppressor role of Gp78.

A recent study reported that cyclin-dependent kinase 5 mediated phosphorylation of Gp78 causes its ubiquitination and degradation, which results in increased rate of neuronal death in animal models of Parkinson’s disease (Wang Y. et al., 2017). Another novel example of Gp78 involvement in neuroprotection is its association with cholesterol homeostasis, which suggests the probable role of Gp78 in slowing down neurodegeneration by maintaining cholesterol metabolism via its well-known ERAD substrate HMG-CoA reductase (Cao et al., 2007; Anchisi et al., 2013; Zhang and Liu, 2015). All these functions of Gp78 in degradation of misfolded forms of different disease-associated proteins and clearance of their inclusion bodies designate this glycoprotein molecule as a QC E3 ubiquitin ligase. Since the identification of RING domain in Gp78 protein and its implication in clearance of multiple target proteins, which crucially regulate several important cellular pathways, interest has generated in finding out other QC roles of Gp78 in amelioration of toxicities generated by aggregation and formation of inclusion bodies by various proteins, associated with diseases. Further work is needed to explore more about its capability to degrade other such proteins. Figure 3A depicts a comprehensive overview of neuroprotective roles of the Gp78 E3 ubiquitin ligase, discovered so far.

## The Multiple Roles of Gp78 in Cellular Proliferation: Novel Insights into Complex Diseases

Discovery, purification, characterization, functional aspect and pathological mechanisms of Gp78 are linked with various types of cancers (Chiu et al., 2008). As we have mentioned above, the protein Gp78 was first identified as surface receptor influencing the metastatic ability of B16-F1 melanoma cells (Nabi and Raz, 1987); still, detailed characterization of its implications in affected pathways and mechanisms in melanoma, as well as different other cancer types, is yet to be accomplished. Purification of acidic and basic AMF from murine protein-free fibrosarcoma reported that metastatic properties of these cells could be affected via AMF-Gp78 signaling (Watanabe et al., 1994). Expression of Gp78 is highly upregulated in bladder carcinoma tissues (Silletti et al., 1993); whereas, in patients with colorectal cancer, immunohistochemical analysis proposed that patients with higher expression of Gp78 have less survival and high risk of cancer recurrence (Nakamori et al., 1994). In prostate cancer cells derived from non-metastatic nude mice (PC-3) and its metastatic variant (PC-3M) mice, expression of Gp78 is differentially upregulated in metastatic conditions (Silletti et al., 1995). The results were again confirmed in a different study based on patients of prostate cancer (Shang and Zhu, 2013).

Esophageal squamous cell carcinoma patients were examined for Gp78 expression and its association with different tumor characteristics; such as, size, growth, invasion and metastasis and again it was found that patients with higher expression of Gp78 have increased risk of cancer with lower survival rate (Maruyama et al., 1995). In patients with transitional cell carcinoma of the bladder, urine samples were tested for AMFR, and 80% of samples were found positive (Korman et al., 1996). Similarly, an upregulated expression of AMFR was reported in cutaneous malignant melanoma (Nagai et al., 1996). All these studies, done by several groups in different cancer types over the years, confirm the elevated expression of AMFR and point towards its possible association with motility and metastasis of cancer cells (Silletti and Raz, 1996). Choriocarcinoma, a cancer of developmental tissues and tissues from oral cell carcinoma further confirmed the association of AMFR with invasiveness and metastasis potential (Niinaka et al., 1996; Yelian et al., 1996). In gastric cancer patients, expression of AMFR reflected poor prognosis and showed a direct correlation with histopathological grades of the tumor (Hirono et al., 1996; Taniguchi et al., 1998).

Expression of Gp78 is also regulated by cell-cell contact under normal conditions, and loss of such a relation is observed during tumor progression (Silletti et al., 1995). A reciprocal relationship between expressions of E-cadherin, a cell adhesion protein and Gp78 has been observed in tissues from bladder carcinomas (Otto et al., 1994), which was later confirmed in another study, where MSV transformed MDCK cell population has been found with lowered E-cadherin and upregulated Gp78 protein expression levels (Simard and Nabi, 1996). This altered E-cadherin/Gp78 ratio could have lethal consequences, as has been reported in patients with bladder carcinomas and gastric cancers, in different studies (Otto et al., 1997; Kawanishi et al., 2000). In lung cancer and thymoma tissues, higher expression of AMFR elevates the risk of tumor progression (Ohta et al., 2000a,b). Higher expression of vascular endothelial growth factor (VEGF) and increased AMFR worsen the disease conditions in patients with non-small cell lung cancer (Kara et al., 2001; Takanami et al., 2001). Similar results were seen in other studies also, where high expression of AMFR was found to be implicated in mediating invasion of lung and oral squamous cell carcinomas and promoting their metastatic capabilities (Niinaka et al., 2002; Takanami et al., 2002; Takanami and Takeuchi, 2003). A positive association between AMFR and metastasis was observed in melanoma cells also (Tímár et al., 2002); whereas, in pulmonary adenocarcinoma patients, AMFR positive subjects have shown a lower post-surgery survival rate, as compared to those having no significant AMFR expression (Kaynak et al., 2005).

Prognostic role of AMF-AMFR complex expression was also identified in human breast cancer by comparative study of breast cancer and non-neoplastic tissues (Jiang et al., 2006). In tongue squamous cell carcinoma and hepatocellular carcinoma (HCC) patients, higher expressions of AMFR, along with Ras homolog family member C (RhoC) and c-met coincides with increased risk of invasion and disease recurrence with low survival (Endo et al., 2006; Wang et al., 2007). Small interfering RNA (siRNA) mediated knockdown and truncated AMFR expression resulted in a decrease in levels of rho-associated coiled-coil containing Protein kinase 2 (ROCK2), cyclin D1 and B-cell lymphoma 2 (Bcl-2), suggesting a possible mechanism, by which AMFR regulates cell cycle and apoptotic pathways (Wang et al., 2015b). Despite several studies indicating the correlation between Gp78 with metastasis in various cancers, the mechanism of how AMFR aids in metastasis was revealed later with identification of Gp78 mediated degradation of metastasis suppressor protein Kangai1 (KAI1), which results in induction of metastasis potential of different cancer cell lines, as well as Gp78-overexpressing transgenic mice (Tsai et al., 2007; Joshi et al., 2010). Identification of another mechanism shed more light on the involvement of Gp78 in metastasis and cell proliferation, where it activates ROCK-2, an important metastasis-associated protein (Wang et al., 2010). Figure 3B summarizes these mechanistic findings, explaining the possible molecules and pathways affected by Gp78, postulating its involvement in transformation, development and progression of tumors.

Contrary to all the above findings, few studies have postulated an inverse correlation between AMFR/Gp78 and tumor progression; for example, microarray expression analysis of bone tumors showed AMFR among downregulated genes in giant cell tumor (Guenther et al., 2005). A recent study on Gp78 null mice showed age-related nonalcoholic steatohepatitis (NASH) and development of HCC proposing the roles of Gp78 in the maintenance of liver homeostasis (Zhang et al., 2015a). Deacetylation of heat-shock protein 5 (HSPA5) by histone deacetylase-6 (HDAC-6) is followed by Gp78-mediated ubiquitination of HSPA5, leading to suppression of invasion and migration in breast cancer cells (Chang et al., 2015). The roles of Gp78 in metastasis and tumor-progression is yet to be fully understood; therefore further research is needed to make a detailed understanding of the molecule so that in future it could be used as a prognostic biomarker of different cancer types and might be exploited for therapeutic purposes.

## Gp78 Can Possess Threshold Or Fault Tolerance: An Unlocking View for Complex Cellular Signaling

The multifaceted functional ability of proteins makes them important macromolecules of the cell. Since the mRNA synthesis, and up to the functional three-dimensional structure formation and appropriate cellular translocation, proteins undergo multifold QC processes (Buchberger et al., 2010; Brandman and Hegde, 2016). Failure at any step leads to protein misfolding, which is directly linked with various neurodegenerative disorders (Hartl, 2017). Components of PQC machinery, including E3 ubiquitin ligases, are commonly found to be associated with more than one pathway in cells to ensure healthy cellular environment (Gestwicki and Garza, 2012).

Interestingly, several crucial E3 ubiquitin ligases such as C-terminus of Hsp70-interacting protein (CHIP), E6AP and Parkin etc. have thoroughly been investigated, and found to be implicated in multiple cellular pathways, and become the cause of incurable diseases when present in non-functional state (Seirafi et al., 2015; Upadhyay et al., 2015; Joshi et al., 2016). Gp78 is another putative E3 ubiquitin ligase that shows similar functional characteristics by having an indispensable association with cellular proteostasis, neuroprotection, and regulation of cell division. The most extensively studied pathway that is critically involved in the maintenance of cellular proteostasis is ERAD, where QC E3 ubiquitin ligases, like Hrd1 and Gp78, provide neuroprotection by reducing the ER stress and maintaining ER homeostasis (Mehnert et al., 2010). In addition to ERAD, Gp78 is involved in various other cellular processes, like cellular signaling, mitophagy induction, immunity and maintenance of metabolic homeostasis, as described in the subsections, to prevent the occurrence of any disease condition.

### Rapid Actions of AMFR as Receptor: Regulating AMF Signaling

AMF is a cytokine that stimulates motility of tumor cells *in vitro* and confers them the lung colonizing ability under *in vivo* conditions (Liotta et al., 1986). As described earlier, the monoclonal antibody 3F3A, directed against AMFR/Gp78 in B16-F1 melanoma cells, possibly binds to AMFR in a manner similar to AMF and thus mounting similar effects, including increased cell motility leading to the identification of Gp78 as the receptor protein for AMF (Nabi et al., 1990, 1992). Further exploration of AMF-AMFR signaling reported that the cellular response for AMF is mediated by phosphorylation of AMFR/Gp78 and production of G-protein and inositol triphosphate (Nabi et al., 1992). Another study observed an increase in expression of AMFR and enhanced cell motility and lung colonizing abilities in murine fibrosarcoma cells in response to the monoclonal anti-Gp78 antibody (mimic effect of AMF), when grown under protein-free cell culture conditions (Watanabe et al., 1993). AMF also acts as an angiogenic factor in human umbilical vein endothelial cells (HUVECs) and affects tumor progression with AMFR in a paracrine manner (Funasaka et al., 2001).

AMF-AMFR signaling in melanoma cells plays a possible role in cytoskeleton rearrangement by activation of small GTPase; it also increases the formation of stress fibers with activation of c-Jun N-terminal kinase (JNK) isoforms and GTPases Rac1 and RhoA, while no significant change in Cdc42 was observed (Tsutsumi et al., 2002). Signaling of AMF-AMFR also induces VEGF signaling by protein kinase C (PKC), and phosphatidyl inositol 3 kinase (PI3K) mediated upregulation of the expression of its receptor Flt-1 on the surface of HUVEC cells (Funasaka et al., 2002). Crystallographic data reveals that C-terminal part of AMF interacts with the extracellular core part of AMFR, and the N-linked glycoside chain of AMFR plays a crucial role in this interaction (Haga et al., 2006). In chondrocytes, AMF and its receptor signaling promotes cell proliferation by increasing expression of pAKT and pSmad2/3; however, it causes a decrease in pSmad1/5 levels (Tian et al., 2015). The importance of AMF-mediated signaling could be understood by the fact that its inhibition leads neuronal cells towards apoptosis, while upregulation has metastatic potential. Therefore, more research is needed to explore the therapeutic aspects of this signaling pathway (Romagnoli et al., 2003).

### AMFR Mediates Selective Mitochondrial Engulfment Via Targeting Mitofusins

Confocal and electron microscopy analyses showed a high degree of association between AMFR tubules of smooth ER, and mitochondria, which is disrupted by high cytosolic levels of the Ca^2+^ ion (Wang et al., 2000; Goetz et al., 2007). Damaged and inactive mitochondria undergo autophagic elimination via selective engulfment of these mitochondria by aggresomes, and the whole process is termed as mitophagy (Lemasters, 2005; Youle and Narendra, 2011). Mitofusins (Mfn1 and Mfn2) are the key factors for mitochondrial fusion and fission machinery, and its parkin-mediated degradation induces mitophagy (Narendra et al., 2008). Gp78/AMFR is another E3 ubiquitin ligase having capabilities to target Mfn1 and Mfn2 for proteasomal degradation, which leads to induction of mitochondrial depolarization and fragmentation in a parkin-independent manner (Fu et al., 2013).

Mitochondria-associated ER localization of Gp78 leads to internalization of AMF via PI3K and dynamin-dependent, non-caveolar, raft-mediated endocytosis (Benlimame et al., 1998). This may lead to inhibition of Gp78-mediated mitofusin degradation, obstructing mitophagy, setting an example of regulation of ERAD by an extracellular ligand (Shankar et al., 2013). Mitofusins regulation inhibits mitochondria-ER interaction, as smooth endoplasmic reticulum (SER)-mitochondria and rough endoplasmic reticulum (RER)-mitochondria contact is controlled by Mfn1 and Mfn2, respectively (Wang P. T. et al., 2015). Further study found that phosphorylation of Gp78 by p38 MAP kinase at S538 prevents degradation of mitofusins and regulate ER-mitochondria interaction (Li et al., 2015).

### Emerging Cellular Functions of Gp78 in Immunity

Exploration of STING signaling pathway, which mounts an innate immune response against foreign DNA of viruses and other microbes led the identification of roles played by Gp78 in the regulation of this pathway (Ishikawa et al., 2009; Shu and Wang, 2014). Observation of antibody responses in adult T-cell leukemia (ATL) patients suggested that AMFR functions as one of the graft-vs.-leukemia (GVL) antigen, involved in evoking antitumor immunity (Hishizawa et al., 2006). ER-associated E3 ubiquitin ligase AMFR and insulin-induced gene 1 (Insig-1) interact with STING on stimulation by viral or microbial DNA and catalyze STING polyubiquitination at K27, which facilitates binding of TANK-binding kinase 1 (TBK 1) and causes translocation of STING to perinuclear microsome to induce an innate immune response (Wang Q. et al., 2014). Gp78 also degrades mitochondrial antiviral signaling (MAVS) protein, which is also known for mounting another kind of antiviral innate immune response by a host cell to increase the production of type-1 interferon (IFN; Jacobs et al., 2014). Therefore, being a membrane protein, Gp78 lies with several other possibilities to detect and evoke downstream signaling pathways against various kinds of pathogenic intrusions.

### Rebooting of Cellular Metabolic Functions by Gp78 E3 Ubiquitin Ligase

Apart from involvement in glucose metabolic pathways, Gp78 stimulation may have positive effects on activation of 12-lipooxygenase and biosynthesis of arachidonic acid metabolite 12 (S)-hydrooxyeicosatetraenoic acid (12-(S)-HETE), which causes alterations in cellular architecture and helps in cell motility (Timar et al., 1993). During cholesterol metabolism, accumulation of sterols accelerates the recruitment of HMG-CoA reductase to ER-bound Insig-1 and -2 proteins, which further form complex with Gp78 to ubiquitinate it in Ufd1-dependent manner and facilitate its proteasomal degradation (Song et al., 2005; Cao et al., 2007; Debose-Boyd, 2008). Liver-specific Gp78 knockout mice have decreased HMGCoR and Insig-1 degradation, which may also cause suppressed levels of sterol-regulatory element binding protein (SREBP), leading to an overall decrease in lipid biosynthesis of the cell, which could be advantageous to patients suffering from metabolic disorders (Liu et al., 2012).

Clinical drugs are mostly metabolized in the liver by ER hemoprotein CYP3A4, which is phosphorylated at different sites (Wang et al., 2012), and hence recognized differentially by two E3 ubiquitin ligases Gp78 and CHIP for its ubiquitination and degradation, under different intrahepatic ubiquitin concentrations (Pabarcus et al., 2009; Wang et al., 2015a). Considering the importance of CYP3A4 in the metabolism of various anticancer drugs, knockdown studies on Gp78 and CHIP to regulate this hemoprotein may provide a therapeutic advantage in anticancer therapies (Kim et al., 2010; Peer et al., 2011).

## Hidden Promising Therapeutic Interventions of Gp78 in Protein Conformational Disorders

**Figure 4:**
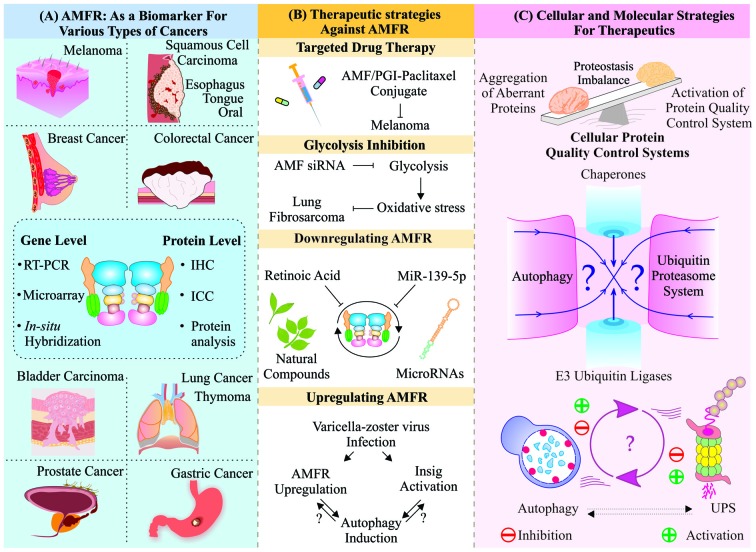
Targeting AMFR/Gp78 for remedial exploration of various maladies: Schematic representation of various approaches developed to utilize multifaceted molecule Gp78 for therapeutic purposes. **(A)** Multiple studies on several cancer types have shown an upregulated expression level of Gp78 proposing it as a crucial molecule to be targeted for further research. **(B)** Few microRNAs (miRNAs) and natural compounds have been used to downregulate Gp78, having a notable delay in metastasis. Targeted therapy and upregulation strategies were also proposed for Gp78 and its ligand autocrine motility factor (AMF). **(C)** Still, targeting a molecule like Gp78, which is known to be involved in multiple pathways, to maintain proteostasis is a delicate function to be accomplished and it further, requires a detailed knowledge of its functional aspects.

Overexpression of AMFR has shown important roles in overall cancer progression, which has been supported by several experimental methods and studies. Based on the reliabilities of these studies, scientists in recent past started proposing AMFR as a prognostic biomarker of tumor development and advanced stages of disease progression, as shown in Figure 4A. Although enough data have accumulated, still acquiring complete knowledge about understanding the overall functional aspects of this protein is underway. Several attempts have also started to modulate the expression or function of AMFR inside the cells to exploit its therapeutic potential in various diseases. Natural compounds, like beta-all-trans-retinoic acid (RA), have been studied to decrease the levels of Gp78 in murine and human melanoma cell lines and to suppress the cell motility (Hendrix et al., 1990; Lotan et al., 1992).

Post-transcriptional suppression strategies, e.g., using microRNAs (miRNAs), were also developed against AMFR to suppress invasion and metastasis. miR-139-5p in colorectal cancer cells and edited miR-376a* in glioblastoma target AMFR showing different effects; while the edited form of miR-376a* leads to increase in AMFR expression, miR-139-5p suppresses metastasis by downregulating AMFR (Choudhury et al., 2012; Song et al., 2014). The tumor cell-specific drug delivery system was developed by conjugation of AMF/PGI-paclitaxel, which get internalized by the raft-dependent endocytic pathway and hence it could be developed as a new therapeutic approach, where AMF acts as a carrier for chemotherapeutic drugs in tumor cells, expressing AMFR under *in vitro* and *in vivo* conditions (Kojic et al., 2008). Blocking the AMF/AMFR signaling pathway could be of significant therapeutic importance, as this may result in downregulation of metastatic abilities of cancer cells (Iiizumi et al., 2008). Using the similar approach, siRNA-mediated downregulation of AMF leads to decreased ability to form tumor mass in human lung fibrosarcoma cells, which might be due to the overall suppression of AMF-mediated signaling (Funasaka et al., 2007).

Based on PCR array analysis, another study on Varicella-zoster virus (VCZ) infected HeLa cells reported increased mRNA levels of AMFR, Insig and BiP, along with upregulated autophagy, whereas several ERAD-associated components were significantly downregulated causing ER stress and unfolded protein response (UPR) inside the cells. Despite the increase in expressions of AMFR and BiP, upregulated UPR and autophagy, and an increase in ER size draw an elusive line between all these components and pathways, which further need to be explored (Carpenter and Grose, 2014). These are the various available therapeutic approaches, which have been tested over the years in the context of medicinal properties of the gene AMFR and its product Gp78 which are chiefly targeted in therapeutics of various cancers. We have represented these findings and their outcomes in Figure 4B for a better understanding.

Like molecular chaperones, QC E3 ubiquitin ligases also have the roles of surveilling the misfolded or accumulated forms of proteins and degrade them through UPS or autophagy (Chhangani et al., 2012). AMFR is a promising receptor molecule with its QC E3 ubiquitin ligase like abilities to sense cellular stresses and mediate appropriate cellular responses to counter the obnoxious changes in cellular proteins (Fang et al., 2001; Shen et al., 2006). As many of Gp78 substrates are components of proteinaceous aggregates, involved in neurodegenerative diseases; it could, therefore, be targeted for therapeutic applications in these diseases (Ying et al., 2009; Yang et al., 2010). Other than its association with protein misfolding-related diseases, it is clinically important for metabolic disorders, as it also maintains homeostatic conditions inside the cells (Zhang et al., 2015a). Despite so many studies on the association of Gp78 with cancer, neurodegeneration and metabolic disorders, the major challenge for researchers and clinicians remains the formulation of a successful therapeutic strategy targeting this gene. Hence, there arises a need to explore Gp78 for its pharmacological significance and drug development in future. In Figure 4C, we have represented an overall view of how various components of cellular PQC machinery coordinate with each other to maintain cellular proteostasis. A delicate balance of these components of PQC machinery is required for a cell to maintain a homeostatic condition. While a slight imbalance may result in several kinds of obnoxious intracellular changes, leading to unwanted disease conditions. Further exploration of functional aspects of QC E3 ubiquitin ligase Gp78 will benefit us in future therapeutic applications of this molecule, which is an indispensable part of multiple cellular pathways.

## Key Questions and Future Perspective

Thirty years since the identification of AMFR as a signaling protein implicated in mediating the metastasis of transformed cells inducing tumor progression, this membrane receptor has been thoroughly investigated later for its many other functions (Nabi and Raz, 1987; Fairbank et al., 2009; Chen et al., 2012). Despite multiple lines of evidence stand by the notion that increased expression of AMFR might have some association with development and maintenance of tumors (Silletti et al., 1993; Otto et al., 1994; Hirono et al., 1996; Korman et al., 1996), still a clear understanding of the mechanism through which AMFR mediates the metastasis and motility of transformed cells is yet to be established. Considering its eminent roles in the progression of several types of tumors, it is now worthy to search out for ways and mechanisms to modulate its activities (Nakamori et al., 1994; Silletti et al., 1995). Several studies have been done to understand the mechanistic part of AMFR regulation, but they have achieved very limited amount of success (Shmueli et al., 2009; Mukherjee and Chakrabarti, 2016). Later years of research has put it into a category of a special class of molecules, called E3 ubiquitin ligases, which led scientists to look for its roles beyond the functions normally played by a membrane receptor protein (Fang et al., 2001). Accumulating literature provides detailed evidence showing that AMFR is crucially implicated in the regulation of cell division, orchestrating ERAD pathway (Song et al., 2005), maintenance of cellular proteostasis (Shen et al., 2006), and mitophagy (Fu et al., 2013). Functions of AMFR also encompasses from mediating innate immune responses against microbe infections (Shu and Wang, 2014) to providing neuroprotection against a variety of cytotoxic insults and several kinds of protein aggregation (Ying et al., 2009).

The importance of AMFR has also increased with establishing knowledge and understanding of its beneficial roles in protection against proteotoxic stresses. Still, much more work is required to elucidate how exactly AMFR E3 ubiquitin ligase bears the burden of maintaining the cellular PQC. It has also been shown that AMFR has a potential to work as an E4 enzyme (Morito et al., 2008). Therefore, there stands a possibility that AMFR might work in conjugation with other E3 ubiquitin ligases to ubiquitinate misfolded or aggregatory proteins. Mechanistic elucidation of such kinds of molecular crosstalk will enhance our understanding about this multipotent signaling protein. Several other QC E3 ubiquitin ligases have already been reported for their potential to interact with molecular chaperones also, to mediate a concerted action against a variety of cellular stresses (McClellan et al., 2005; Upadhyay et al., 2015).

Although the indispensable association of AMFR with ERAD pathway has been thoroughly reported, where, in association with other ER membrane-bound E3 ubiquitin ligase complexes and ER resident chaperones, AMFR clears proteotoxic load of unfolded or misfolded protein species generated inside this cellular compartment due to several types of extra-and intracellular stresses. Multiple studies have also provided crucial insights into neuroprotective roles of AMFR; still, more work is needed to understand the applicability of this protein for consideration as a possible future therapeutic target against multiple neurodegenerative diseases. Roles of this E3 ubiquitin ligase in the maintenance of healthy cellular proteome by selectively targeting its substrate proteins for proteasomal degradation, is an area to explore further for developing a better understanding of the therapeutic potential of AMFR gene.

## Author Contributions

VJ and AU executed complete drawing of figures. AK provided critical inputs. AM formulated the entire concept of the manuscript and designed the initial draft of figures. All authors reviewed the manuscript.

## Conflict of Interest Statement

The authors declare that the research was conducted in the absence of any commercial or financial relationships that could be construed as a potential conflict of interest.

## Abbreviations

ALS: Amyotrophic lateral sclerosis
AMF: Autocrine motility factor
AMFR: Autocrine motility factor receptor
ATL: Adult T-cell leukemia
Bcl-2: B-Cell CLL/Lymphoma 2
BHK-21: Baby hamster kidney-21
Bip: Immunoglobulin heavy chain-binding protein
CFTR: Cystic fibrosis transmembrane conductance regulator
CHIP: Carboxy Terminus Of Hsp70-Interacting Protein
CMT: Charcot-Marie-Tooth
CUE: Coupling of ubiquitin conjugation to the ER degradation
CYP3A4: Cytochrome P450s of the 3A subfamily 4
DGAT2: Diacylglycerol acyltransferase isoform 2
ER: Endoplasmic reticulum
ERAD: Endoplasmic reticulum associated degradation
ESCC: Esophageal squamous cell cancer
G2BR: Ube2G2 binding region
Gp78: Glycoprotein 78
GVL: Graft-vs.-leukemia
HCC: Hepatocellular carcinoma
HDAC-6: Histonedeacetylase-6
HECT: Homologous to The E6-AP Carboxyl Terminus
HERP: Homocysteine-induced ER protein
HMGCoA: 3-hydroxy-3-methylglutaryl coenzyme A
Hrd1: HMG-CoA Reductase Degradation 1 Homolog
HSPA5: Heat-shock protein 5
HSV 1: Herpes simplex virus 1
htt: Huntingtin
HUVECs: Human umbilical vein endothelial cells
IFN: Interferon
Insig-1: insulin-induced gene 1
JNK: JUN N-Terminal Kinase
KAI1: Kangai 1
LDL: Low-density lipoproteins
MAVS: Mitochondrial antiviral signaling
Mfn: Mitofusin
MGRN1: Mahogunin Ring Finger 1
miRNAs: Micro RNAs
MSV-MDCK: Moloney sarcoma virus (mos)-transformed MDCK
MVBs: Multivesicular bodies
NASH: Nonalcoholic steatohepatitis
NSCLC: Non-small cell lung cancers
OS: Oligomerization site
PHD: Plant Homeodomain
RhoC: Ras homolog family member C
PI3K: Phosphatidylinositol 3 kinase
PKC: Protein kinase C
PMP22: Peripheral myelin protein 22
PQC: Protein quality control
PrP: Prion
QC: Quality control
RA: Retinoic acid
RER: Rough endoplasmic reticulum
RING: Really Interesting New Gene
RMA1: Ram 1 homolog
RNF45: RING finger protein 45
ROCK-2: Rho Associated Coiled-Coil Containing Protein Kinase 2
SCC: Squamous cell carcinoma
SER: Smooth endoplasmic reticulum
SOD-1: Superoxide dismutase-1
SREBP: Sterol-regulatory element binding protein
STING: Stimulator of interferon genes
SVIP: Small VCP interacting proteins
TBK 1: TANK binding kinase 1
TG: Triacylglycerol
TRIM25: Tripartite Motif Containing 25
Ufd1: Ubiquitin fusion degradation 1
UPR: Unfolded protein response
UPS: Ubiquitin proteasome system
VCZ: Varicella-zoster virus
VEGF: Vascular endothelial growth factor
VEGFR: Vascular endothelial growth factor receptor
VIM: p97/VCP (valosin containing protein) interacting motif
VLDL: Very low-density lipoproteins.
